# When stripes in clothes deceive: Cross-cultural examination of perceptual and belief discrepancies about horizontal stripes in clothes

**DOI:** 10.1371/journal.pone.0347495

**Published:** 2026-04-30

**Authors:** Antonis Koutsoumpis, Elias Economou, Zai-Fu Yao, Erik Van der Burg

**Affiliations:** 1 School of Business and Economics, Vrije Universiteit Amsterdam, Amsterdam, Netherlands; 2 Department of Psychology, Lab of Experimental Psychology, University of Crete, Rethymno, Greece; 3 College of Education & Research Center for Education and Mind Sciences, National Tsing Hua University, Hsinchu, Taiwan; 4 Section Clinical Developmental Psychology, Vrije Universiteit Amsterdam, Amsterdam, Netherlands; University of Bologna Department of Psychology #39;Renzo Canestrari#39;: Universita degli Studi di Bologna Dipartimento di Psicologia Renzo Canestrari, ITALY

## Abstract

Despite the common belief that horizontal stripes in clothes make someone look wider, behavioral studies show that horizontally striped stimuli are perceived as thinner compared to equally wide non-striped stimuli. In the present study, we examined the extent to which participants’ beliefs regarding the thinning or widening effect of horizontal stripes were related to their visual perception of horizontally striped versus neutral stimuli in a behavioral task. Data were collected across three countries to explore possible cross-cultural generalizability of the phenomenon. In Experiment 1 (*n* = 316; Greece, Netherlands), we measured participants’ beliefs regarding the effect of horizontal stripes in clothes, verifying the popular belief that horizontal stripes are, indeed, thought to have a widening effect regardless of country. In Experiment 2 (*n* = 419; Greece, Netherlands, Taiwan), participants self-reported their beliefs regarding the effect of horizontal stripes and, also, completed a behavioral task comparing the width of a striped and non-striped dress. The results showed that (a) in line with previous research, the horizontally striped dress was perceived as thinner compared to the non-striped dress, and (b) the more participants believed that horizontal stripes make someone look thin, the more they perceived the striped dress as thinner. However, the relation between beliefs and size perception was non-significant for participants who believed that horizontal stripes make someone look wider. No cross-cultural differences were found for this asymmetrical effect, highlighting the universality of the findings.

## Introduction

Horizontal stripes in clothes have a long history. In medieval times they were considered a sign of mystery and sorcery, in the 20^th^ century they were the dress code for inmates and, today, stripes are a choice of style and fashion [1–3]. Interestingly, it seems that people hold certain preconceptions about the perceptual effect of horizontal stripes, and it seems to be generally believed that horizontal stripes make someone look wider (e.g., [4]).

The available quantitative evidence in support of the popular belief seems to come, surprisingly, from a single study. In a footnote, [5] reported that in a sample of 31 Psychology students, “*58% stated [self-reported] that horizontal stripes have a fattening effect, 26% stated that horizontal stripes have a thinning effect, and 16% stated that horizontal stripes have neither effect*” (p. 755). Even though this seems to be the first quantitative report in support of the popular belief, the study was limited by the small, non-representative sample, predominantly comprised of women Psychology students. In the present study, we quantify people’s beliefs regarding the effect of horizontal stripes in clothes, in the general population (Experiment 1).

### Helmholtz illusion and personal beliefs

The popular belief that horizontal stripes in clothes make someone look wider comes at sharp contrast with research findings consistently showing that clothes with horizontal stripes make someone look thinner compared to equally wide clothes with either vertical stripes [6,7] or no stripes at all [5]. The thinning effect of horizontal stripes was originally described by Helmholtz [8], who showed that a square filled with horizontal stripes appeared taller (thus, thinner) compared to an equally wide square filled with vertical stripes; an optical illusion known as the ‘Helmholtz illusion’.

The Helmholtz illusion has been explained based on Gestalt Psychology [9] suggesting that even though the *local* orientation of the stripes is horizontal, their *global* orientation is perceived to be vertical (i.e., the horizontal stripes are perceived to be ‘stacked’ on top of each other, that is, grouped in a vertical manner). As the global orientation of the stripes dominates their local orientation, stimuli with horizontal stripes are perceived as taller and, thus, thinner. More recently, [10] suggested that attentional mechanisms might also explain the illusion. They showed that by manipulating participants’ attention either in the same or the opposite direction of the illusion (i.e., inducing a widening or a thinning effect) it is possible to amplify or reduce the perceptual effect.

Whereas Helmholtz was the first to report the thinning effect of horizontal stripes in geometrical shapes (using squares), more recent research has reported similar effects using striped clothes. Behavioral studies consistently show that clothes with horizontal stripes are perceived as making someone look thinner compared to equally wide clothes with vertical stripes, either in two- or three-dimensional stimuli [7]. The thinning effect of horizontal stripes seems to diminish as the body size increases [6] and, in large body sizes, the effect seems to reverse ([11] as reported by [6]; original paper in Japanese). However, when directly comparing stimuli with horizontal versus vertical stripes, it is impossible to disentangle whether the thinning effect is attributed to a thinning effect of horizontal stripes or to a widening effect of vertical stripes (or both). [5] showed that the thinning effect of horizontal stripes is observed even when clothes with horizontal stripes are compared to equally wide clothes with no stripes at all (i.e., a neutral condition), with the thinning effect being independent of the luminance of the stimuli themselves or that of the background. Thus, horizontal stripes seem to have an independent illusory effect that makes horizontally striped stimuli look thinner compared to equally wide non-striped stimuli.

Interestingly, despite the fact that horizontal stripes are perceived as thinner compared to non-striped stimuli, participants who perceived horizontally striped stimuli to be thinner compared to equally wide non-striped stimuli, self-reported they believed that horizontal stripes made someone look wider [5]. This is a counter-intuitive finding, as one would expect that participants’ perception would align with their beliefs, self-reporting that horizontally striped stimuli have a thinning effect. No explanation has been provided for the discrepancy between participants’ beliefs and their size perception, nor has it been explored whether participants’ self-reported beliefs are related to their perceptual judgments. This omission is significant because, based on previous research, one could expect that someone’s beliefs might affect perceptual processes, such as size perception [12–14].

Even though clothes with horizontal stripes are perceived as thinner compared to equally wide clothes with vertical or no stripes [5–7], not all studies report a thinning effect (i.e., [4,15]). In a study comparing images of participants wearing clothes with various patterns [4], the authors reported that horizontally striped clothes were perceived as neither wider nor thinner compared to bright clothes, whereas in one out of their two studies, horizontally striped clothes were perceived as wider compared to darker clothes. However, as their stimuli were scraped from the internet, they were non-standardized and the large number of confounding variables (e.g., body shape, fit of clothes, size of stripes, luminance, lighting, camera angle, facial information, emotional state) that were not statistically or methodologically controlled for, undermined the credibility of the findings. For instance, the stimuli were overrepresented by individuals who were classified as overweight (25%) or obese (56%). Body size is an important confounding variable, as previous studies suggest that as body size increases, the thinning effect of horizontal stripes seems to diminish [6] or reverse [11].

In another study [4], the authors reported that a female confederate wearing a horizontally striped dress was perceived as having larger body weight, compared to (the same confederate) wearing a vertically stiped or a non-striped dress. However, the study was limited in that participants provided their responses retrospectively based on recollection, instead of judging the stimuli in real time, as the behavioral task was taking place. Based on the present results of Experiment 1, showing that the majority of people seem to believe that horizontal stripes make someone look wider, the design of [4]’s study likely captured participants’ pre-existing beliefs instead of size perception. Overall, based on previous behavioral studies reporting a thinning effect of horizontally striped dresses, in the present study we hypothesized that a horizontally striped dress would be perceived as thinner compared to an equally wide dress without stripes.

#### Cross-cultural generalization.

As the belief regarding the effect of horizontal stripes in clothes has been mainly populated in Western cultures, to get a better idea about the possible cross-cultural generalization of the phenomenon, in the present study we compared Western and non-Western samples, specifically two European (Greece, the Netherlands) and one East Asian (Taiwan) countries. Naturally, this is not an exhaustive culture comparison, but it will allow us to test the possible universality or culture-specificity of the phenomenon between two large cultural groups, namely Western (typically characterized as higher in individualism) and Eastern (typically characterized as higher in collectivism). Beauty standards differ significantly between Western and Eastern cultures [16–19], thus participants’ beliefs about the effect of horizontal stripes in clothes might differ. More importantly, extensive cross-cultural research suggests that participants from collectivistic cultures characteristically employ more context-sensitive, holistic perceptual strategies compared to participants from Western cultures [20,21]. Such holistic processing enhances sensitivity to background cues, which might amplify or attenuate size illusions depending on cultural standards [22]. As a result, the present study will allow us to test the extent to which the popular belief regarding horizontal stripes in clothes, as well as its possible effect on size perception, generalizes across cultures.

In summary, in the present study we conducted two cross-cultural experiments. In Experiment 1, we documented people’s beliefs regarding the effect of horizontal stripes in clothes in a representative sample of the general population. In Experiment 2, we explored whether people’s beliefs regarding the effect of horizontal stripes in clothes were correlated with their size perception of a horizontally striped (vs. neutral) stimulus in a behavioral matching task. To explore the cross-cultural generalizability of the results, data were collected across two Western (Greece, Netherlands) and one East Asian cultures (Taiwan; only in Experiment 2). In the remaining of the manuscript, the focus will be on the effect of horizontal – instead of vertical – stripes and, hence, the word ‘stripes’ will refer to horizontal stripes, except otherwise specified.

## Experiment 1

The aim of Experiment 1 was to quantify people’s beliefs regarding the effect of horizontal stripes in clothes in a representative sample of the general population. For exploratory reasons we also collected information about participants’ beliefs regarding the effect of vertical stripes in clothes, as well as bright and dark clothes. To get a better idea whether the popular belief held across different countries, data were collected across two European countries, namely Greece and the Netherlands (due to resource constraints we did not collect data in Taiwan).

As the belief that horizontal stripes make someone look wider is incongruent with previous empirical findings employing size perception tasks [5–7], people who hold this belief might also hold erroneous beliefs about other empirical findings. Such erroneous beliefs are known as ‘neuromyths’, that is, common misconceptions about the human brain that are not supported by empirical evidence, such as that we only use 10% of our brain, or that people learn better when they receive teaching in their preferred learning style [23]. Therefore, as an extra exploratory step, we also aimed to measure the possible relation between neuromyths with participants’ beliefs on the effect of horizontal stripes in clothes.

### Materials and methods

#### Sample and procedure.

Participants were recruited through the online crowd sourcing panel Prolific [24]. We collected data from *n* = 316 participants (158 men, 154 women, four non-binary; 30.78 years old, *SD* = 9.90), half of whom were from Greece and half from the Netherlands. The experiment lasted approximately 15 minutes and participants were compensated with €2.20. The experiment was approved by the ethical boards (VCWE-2021–173) and participants were free to consent (in written electronic form) to participate after receiving information about the experiment. Data were collected on the 25th of September 2023.

#### Translation.

The experiment was originally developed in the English language. Translation into Greek and Dutch followed the committee approach [25]. More specifically, the material was independently translated into the target languages (i.e., Dutch, Greek) by two translators native in the target language and fluent in English. Then, the two translators compared their translations until they reached a common final version.

#### Measures.

*Beliefs*. Personal beliefs about the effect of horizontal stripes in clothes were measured using a single item: “I think that horizontal stripes (≡) in clothes make someone look:” with participants indicating their responses in a continuous slider, ranging from 0 to 100 (0 = *Much thinner*; 50 = *Neither thinner nor fatter*; 100 = *Much fatter*). For exploratory reasons, we also asked participants to indicate the effect of vertical stripes in clothes (“I think that vertical stripes (⦀) in clothes make someone look:”), as well as the effect of bright (“I think that bright clothes make someone look:”) and dark (“I think that dark clothes make someone look:”) clothes. Finally, again for exploratory reasons, we asked participants to indicate their cultural beliefs about the effect on body size of horizontal and vertical stripes, as well as bright and dark clothes. Cultural beliefs were operationalized as the extent to which participants believed that *other* people hold certain beliefs. For instance, the cultural (instead of personal) belief about the effect of horizontal stripes in clothes read: “I think that most *other* people believe that horizontal stripes (≡) in clothes make someone look:”. Responses were given on the same 100-point scale, as described above. The eight items (four measuring personal beliefs, four measuring cultural beliefs) were presented in random order.

*Neuromyths.* We measured belief in neuromyths using the scale developed by [26]. The scale has 22 items that measure three dimensions: Neuromyths (11 items; e.g., “We only use 10% of our brain”), Cognitive enhancement (5 items; e.g., “Playing commercial video games improves cognitive performance”), and Brain facts (6 items; e.g., “We use our brains 24h a day”). Participants indicated the extent to which they agreed with each statement using a continuous slider ranging from 0 to 100 (0 = Strongly disagree, 100 = Strongly agree). The 22 items were presented in random order.

*Body size and type, height, and weight*. We measured participants’ body size and type using the BODy Size and Shape (BODSS) scale ([27]; Figs 2-3). Participants indicated among seven images the image that best described their body size and type (there is a different set of images for men and women). This information was later recoded into four categories, namely Underweight, Normal weight, Overweight, and Obese, following the recommendations by [27]. We also asked participants to indicate their height and weight.

**Fig 1:**
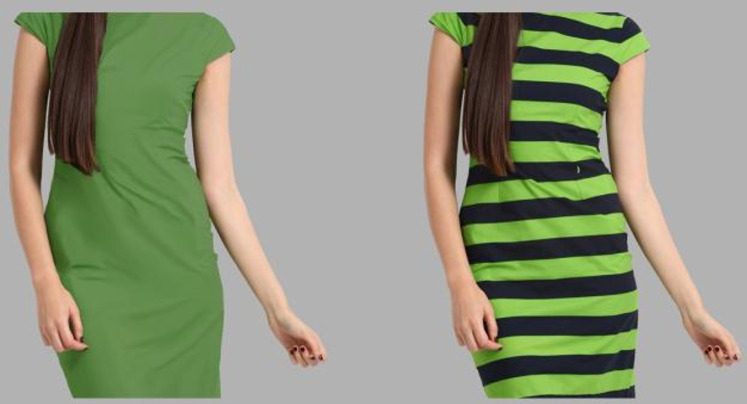
Image for the task regarding what dress participants would advise a friend to buy to look thinner (Experiment 1); the same stimuli were also used in Experiment 2.

*Extra variables*. To get a better picture of participants’ views, we asked to indicate the number of clothes with horizontal stripes they owned (*Mean* = 4.15; *SD* = 8.73), the degree to which they were interested in fashion (100-point slider; 0 = Not at all; 100 = Care a lot; *Mean* = 49.92; *SD* = 28.13), the degree to which they care about the way they look when they dress up (100-point slider; 0 = Not at all; 100 = Care a lot; *Mean* = 58.99; *SD* = 23.61), and the degree to which they take into account the possible thinning (or widening) effect of stripes when selecting clothes (100-point slider; 0 = Not at all; 100 = A lot; *Mean* = 31.03; *SD* = 30.54). Finally, we presented participants with the following scenario and asked them to respond using their gut feeling: “*A friend tries to decide between one of the two dresses in the pictures below* [Fig 1]*. She likes both dresses equally but she wants to select the dress that will make her look thinner. Her body size and type are similar to the model in the pictures, and the size of the dresses is exactly the same in both pictures. What dress would you advise her to buy?*”. Responses were given on a 100-point slider (0 = Definitely the non-striped dress; 50 = It makes no difference; 100 = Definitely the striped dress; *Mean* = 43.66; *Median* = 50; *SD* = 29.2). We also included an open-ended question asking participants to provide a brief rationale for their decision.

### Results

Regarding the Neuromyths scale, we performed a principal component analysis to explore whether the three dimensions (Neuromyths, Cognitive enhancement, Brain facts) loaded on a single or multiple components. The results showed that the three dimensions loaded on a single principal component which explained 54.32% of the variance. Thus, we calculated a ‘total’ Neuromyth score, taking the average of the three dimensions, with higher scores indicating stronger belief in Neuromyths. Across both languages, the Cronbach’s alpha across the 22 items was 0.72, and the mean score for the overall Neuromyth scale was 49.03 (*SD* = 9.74).

There were no significant differences between the two countries, except for the Dutch sample scoring somewhat higher in Neuromyth beliefs (*t* = 3.23, *p* = .001; Greece = 47.28, Netherlands = 50.77) suggesting stronger Neuromyth beliefs, as well as Dutch participants being taller than Greek participants (*t* = 2.35, *p* = .02; Greece = 172.8 cm, Netherlands = 175.4 cm). As a result, in Table 1 we present the descriptive statistics for the pooled sample. The most important findings were that participants believed that clothes with horizontal stripes make someone look wider (*Mean* = 64.37, *SD* = 15.68), providing empirical support to the popular belief.

**Table 1:** Descriptive statistics averaged across the Netherlands and Greece.

Variable	Mean	*SD*
Age	30.78	9.90
Horizontal stripes beliefs (personal)	64.37	15.68
Horizontal stripes beliefs (cultural)	67.59	18.55
Vertical stripes beliefs (personal)	39.62	16.27
Vertical stripes beliefs (cultural)	35.25	18.19
Dark clothes beliefs (personal)	33.38	16.41
Dark clothes beliefs (cultural)	29.71	17.70
Bright clothes beliefs (personal)	53.72	13.63
Bright clothes beliefs (cultural)	56.68	16.17
Neuromyths	49.03	9.74
Height	174.16	9.79
Weight	75.07	17.42
BODDS	2.27	0.74
Number of striped clothes	4.15	8.73
Importance of fashion	49.92	28.13
Importance of outlook	58.99	23.61
Stripe consideration	31.03	30.54
Friend advice	43.66	29.20

*Notes*. BODDS = body type and shape scale; for stripes (and clothes’ brightness) beliefs: Values 0–49 = thinning effect; 50 = Neither thinning nor widening; 51–100 widening effect.

**Fig 2:**
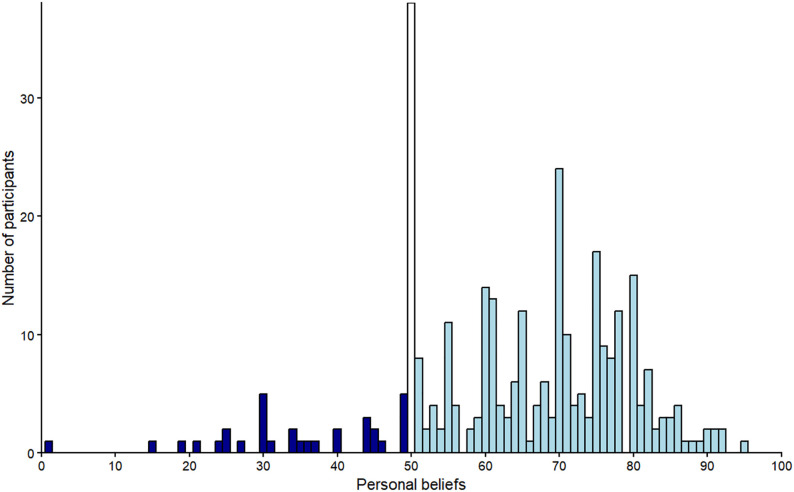
Experiment 1 – Histogram of participants’ beliefs regarding the effect of horizontal stripes. *Notes*. Values ≤ 49 (dark blue bars) indicate that horizontal stripes in clothes make someone look thinner, with smaller values indicating a more pronounced thinning effect; the value 50 (white bar) indicates neither a fattening nor a thinning effect; values ≥ 51 (light blue bars) indicate a widening effect, with larger values indicating a more pronounced effect*.*

Fig 2 illustrates the distribution of participants’ beliefs regarding the effect of horizontal stripes. We noted that participants’ beliefs regarding clothes with horizontal stripes followed a trimodal instead of a normal distribution. A first group (*n* = 32; values 0–49, *Mean* = 34.59, *SD* = 11.54) believed that horizontal stripes make someone look thinner; a second group (*n* = 38; value 50) believed that horizontal stripes have neither a widening nor a thinning effect; a third group (*n* = 246; values 51–100, *Mean* = 70.46, *SD* = 10.69) believed that horizontal stripes have a widening effect. That is, the majority of participants (77.85%) believed that horizontal stripes in clothes make someone look wider, instead of thinner (10.13%). The results were similar (not significantly different) in both countries, verifying the popular belief in the general population.

Table 2 presents the intercorrelations among all variables. We also present correlation coefficients for the subgroups of participants who believed that stripes make someone look thinner or wider. However, as the sample size for the subgroup of participants who believed that horizontal stripes make someone look thin was small (*n* = 32), we will mainly discuss the results for the entire sample. The most notable findings were that participants who believed that horizontal stripes make someone look wider also had stronger Neuromyth beliefs (*r* = 0.15), were somewhat older (*r* = 0.18), were less likely to own striped clothes themselves (*r* = −0.15), were taking more into consideration the possible widening effect of horizontal stripes when buying clothes (*r* = 0.27), and were more likely to suggest to a friend to buy a non-striped dress instead of an equal in size striped dress (*r* = −0.26). In short, the results of Experiment 1 showed that the majority of the general population indeed believed that clothes with horizontal stripes make someone look wider, and this belief was positively correlated with beliefs in neuromyths.

**Table 2:** Experiment 1 – Intercorrelations among main variables for the combined dataset (Greece, Netherlands)*.*

	*M*	*SD*	1	2	3	4	5	6	7	8	9	10	11	12	13
1. Belief horizontal stripes (thin)	34.59	11.54													
2. Belief horizontal stripes (wide)	70.46	10.69													
3. Belief horizontal stripes	64.37	15.68													
4. Neuromyths	49.03	9.74	−.05	.12^†^	0.15^**^										
5. Gender	1.51	0.53	−.11	.08	0.15^**^	−0.03									
6. Age	30.78	9.90	−.06	.34^***^	0.18^**^	0.21^***^	−0.01								
7. Height	174.16	9.79	.26	−.02	−0.08	0.04	−0.69^***^	−0.01							
8. Weight	75.07	17.42	.19	.06	0.01	0.10	−0.45^***^	0.23^***^	0.54^***^						
9. BODDS	2.27	0.74	.12	.06	0.10	0.11^*^	0.04	0.25^***^	−0.06	0.57^***^					
10. Number of striped clothes	4.15	8.73	−.24	−.09	−0.15^**^	0.10	0.06	0.02	−0.03	−0.06	0.00				
11. Fashion importance	49.92	28.13	−.26	.01	0.05	0.03	0.36^***^	−0.19^***^	−0.21^***^	−0.25^***^	−0.12^*^	0.08			
12. Outlook importance	58.99	23.61	−.11	−.01	0.07	0.14^**^	0.24^***^	−0.11^*^	−0.17^**^	−0.15^**^	−0.01	0.03	0.58^***^		
13. Stripe consideration	31.03	30.54	.16	.23^***^	0.27^***^	0.18^***^	0.22^***^	0.15^**^	−0.19^***^	0.07	0.17^**^	−0.02	0.24^***^	0.26^***^	
14. Friend advice	43.66	29.20	−.14	−.19^**^	−0.26^***^	0.01	−0.04	0.01	0.01	0.01	−0.03	−0.05	−0.10	−0.13^*^	−0.13^*^

*Notes*. *N* = 316; ‘thin’ (*n* = 32) refers to the sub-group of participants who believed that horizontal stripes make someone look thinner; ‘wide’ (*n* = 246) refers to the sub-group of participants who believed that horizontal stripes make someone look wider; *M* = Mean; *SD* = Standard deviation; regarding gender: men = 1, women = 2; BODDS = body size and shape scale. ^†^*p* < .06; ^*^p < .05; ^**^p < .01; ^***^p < .001.

## Experiment 2

The aim of Experiment 2 was to explore whether beliefs correlated with participants’ perceptual (thinning) effect. Previous studies show that participants’ expectations are positively related to taste [13] or size perception [12], with perception being in line with their expectations ([14]; but see [28] for counter-findings). Even though we are not aware of any studies reporting that participants’ beliefs and perception are negatively related, we refrained from hypothesizing the direction of the relation between participants’ beliefs and size perception of horizontal stripes. Instead, we were interested in quantifying a possible correlation between beliefs and perception. The logical possibility of a negative relation – the more participants believe that stripes make someone look wider the stronger the perceptual *thinning* effect will be – seemed less plausible to us. To explore possible cross-cultural generalizability, we collected data from Greece, Netherlands, and Taiwan.

Regarding size perception, some previous studies have employed a forced-choice response format, asking participants to select the stimulus that looked either thinner or wider [7,10]. Such response format has been criticized for response bias [29–31] as it forces participants to select one of two choices, which might make it difficult to disentangle perceptual effects from participants’ expectations or beliefs. To avoid this pitfall, in the present study we employed a perceptual matching task (adjustment task), asking participants to adjust the size of one stimulus as necessary, until they perceive it to be equal in size with another stimulus. Adjustment tasks give participants direct control over stimuli comparison making it more difficult to favor one stimulus over the other, minimizing the possibility that the task itself is intertwined with participants’ beliefs (compared to forced-choice paradigms; see [5] for a similar approach).

### Materials and methods

#### Sample and procedure.

*Participants.* We collected data from *n* = 428 Psychology students. After excluding participants who failed to pass the attention check (*n* = 9), the final sample size consisted of *n* = 419 participants (Greece *n* = 163; Netherlands *n* = 129; Taiwan *n* = 127). There were 69 men, 347 women, two non-binary, one did not disclose gender, and participants were on average 21.01 years old (*SD* = 4.55). The experiment lasted approximately 30 minutes and participants received course credits for their participation. The experiment was approved by the ethical boards (VCWE-2021–173) and participants were free to consent (in written electronic form) to participate after receiving information about the experiment. Data were collected between the 21^st^ of November 2022 and 29^th^ of November 2024.

#### Translation.

The tasks and instructions of the experiment were translated in Greek and Chinese following the same approach as in Experiment 1. The Dutch sample completed the task in English.

#### Perceptual task.

*Stimuli and apparatus*. The perceptual task was programmed in the open-source software OpenSesame version 3.3.7 [32]. Participants sat in a dimly lit cubicle approximately 72 cm from a 22-inch monitor with resolution 1,680 × 1,050 pixels and provided their responses in a standard QWERTY keyboard. Across every trial, participants saw the images of two female figures, one dressed in a striped dress and the other in a non-striped dress, against a plain, light-grey background (see Fig 1 for an example). As the monitor luminance varied somewhat across the labs of the three countries, after considering multiple stimuli options of different luminance, we chose a pair of stimuli (striped dress, non-striped dress) that had almost identical luminance across all labs. The luminance of the grey background was 92 cd/m^2^. The luminance of the two dresses (average luminance around the belly area) was approximately identical (46 cd/m^2^) to ensure that any thinning or fattening effect could not be accounted for by luminance differences (see [5]).

The two stimuli had fixed height (18.75° visual angle) and their width (measured as the maximum distance between stimulus’ left-right hand) was 15.81°. When making comparisons, the width was allowed to vary from 13.20° to 18.41°, in steps of 0.087°. The stimulus that served as the comparison (standard) was always set on its original size (15.81°), and the stimulus that participants needed to adjust (deviant) was always randomly determined in a range of values (in steps of ±1.73°) from its original size, to avoid a hysteresis effect. Each stimulus was located to the right and left of the center of the screen, respectively (distance at 9.11°), whereas the *x* and *y* coordinates were randomly jittered (between ±0.43°). Each stimulus (striped and non-striped) was randomly presented to the left and right side of the screen, with equal chances.

*Procedure and design*. Participants’ task was to adjust (increase or decrease) the width of one of the two stimuli (deviant) until they perceived it to be equally wide with the width of the other stimulus (standard). Participants could adjust the size of the stimulus using the *up* and *down* arrow keys in the keyboard and provide their final response by pressing the *spacebar*. At the beginning of each trial, a word cue (presented for 500 ms) informed participants which of the two stimuli they needed to adjust (i.e., the striped or non-striped dress). There were 60 trials in total, in half of which participants needed to adjust the striped and in the other half the non-striped dress. The stimulus that needed to be adjusted each time was randomly determined, and participants had no time limit to submit their responses. The dependent variable was the difference in perceptual size between the standard stimulus and the deviant stimulus, and it was operationalized as the point of subjective equality (PSE). For instance, if the standard stimulus was the non-striped dress and the deviant stimulus the striped dress, a positive PSE indicated a thinning effect, as participants needed to increase the width of the horizontally striped dress to perceive it as equal in size with the non-striped dress. Before the beginning of the task, participants were presented with instructions regarding the task and practiced a few trials.

#### Measures.

After the perceptual task, participants completed the same measures as in Experiment 1. That is, the questionnaire about their beliefs of the effect of horizontal stripes in clothes (but also vertical stripes, dark, and bright clothes), the neuromyth scale, control variables, and demographic information. The Cronbach’s alpha for the Neuromyths scale was.72 for the entire sample.

#### Statistical plan.

Unlike a standard multiple regression analysis, which assumes that all observations are independent and belong to a single level of analysis, in our data this assumption was violated. This was because the repeated PSE measurements were nested within participants, and participants were nested within countries. Therefore, we employed a linear mixed-effects (multilevel) model which explicitly accounts for this hierarchical structure by modeling both fixed effects (e.g., beliefs, belief group, and control variables) and random effects associated with higher-level units (e.g., participants and country). The data were analyzed using the R package *lme4* [33]. The dependent variable was PSE (Level 1) measured as visual angle (^o^). PSE was coded such that positive values indicated a thinning effect, and negative values indicated a widening effect. The independent variable was the personal beliefs regarding the effect of horizontal stripes (Level 2). We also controlled for the variables of neuromyths, gender, age, weight, height, body size and shape (Level 2), as well as country (Level 3). Before entering the linear mixed-effects model, all control and predictor variables were mean-centered based on their relevant groups. Specifically, neuromyths and age were centered within country by subtracting each country’s mean from individual scores. Height, weight, and BODDS were centered within country and gender, such that values reflected deviations from the mean of participants of the same gender within the same country. Self-reported beliefs on the effect of horizontal stripes were centered per country and belief group (see below), so that scores represented deviations from the corresponding group mean. We applied centering (rather than standardization) to remove between-country mean differences while preserving the original metric and variance of the measures for interpretability. In the Results section we report the main findings of our analysis, and on the OSF page of the project we provide the R script with the detailed step-by-step linear mixed-effects model analysis.

#### Excluded observations.

We excluded trials that were classified as outliers by at least half of three approaches based on a composite outlier score using the R package *performance* [34,35]. These approaches included a univariate model (*z*-scores robust; [36]), a multivariate model (Minimum Covariance Determinant; [37]), and a model-based approach (Cook’s distance; [38]). The trial-based exclusion analysis was based on the following variables: PSE, personal beliefs about horizontal stripes, log-transformed response time per trial, and condition. This approach excluded 87 trials (out of 25,740). We also excluded non-binary participants (*n* = 3), as this group was small for any meaningful statistical analysis related to gender.

### Results

#### Descriptive statistics.

**Fig 3:**
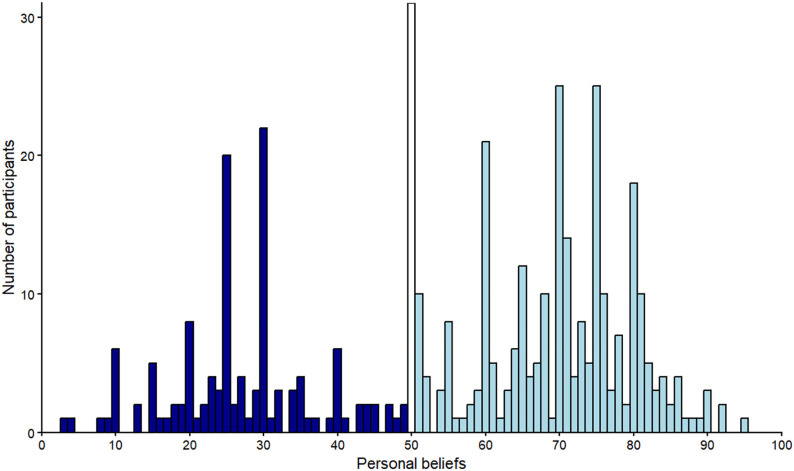
Experiment 2 – Histogram of participants’ beliefs regarding the effect of horizontal stripes. Notes. Values ≤ 49 (dark blue bars) indicate that horizontal stripes in clothes make someone look thinner, with smaller values indicating a more pronounced thinning effect; the value 50 (white bar) indicates neither a fattening nor a thinning effect; values ≥ 51 (light blue bars) indicate a widening effect, with larger values indicating a more pronounced effect.

Similar to Experiment 1, we noted that participants’ beliefs followed a trimodal instead of a normal distribution, representing three different groups (see Fig 3). A first group (*n* = 125; values 0–49, *Mean* = 27.06, *SD* = 9.83) believed that horizontal stripes make someone look thinner; a second group (*n* = 31; value 50) believed that horizontal stripes have neither a fattening nor a thinning effect; a third group (*n* = 263; values 51–100, *Mean* = 71.03, *SD* = 10.39) believed that horizontal stripes have a fattening effect. Unlike Experiment 1, in Experiment 2 the subgroups of participants who believed that horizontal stripes have a thinning or widening effect were more balanced, even though approximately two thirds of participants (62.77%) believed that horizontal stripes in clothes make someone look wider instead of thinner (29.83%).

Table 3 presents the intercorrelation across all variables of Experiment 2. Regarding participants’ beliefs about horizontal stripes, we also calculated the correlations for the sub-groups of participants who believed that horizontal stripes make someone look thinner or wider. The most notable findings were that PSE was significantly correlated with participants’ beliefs regarding the effect of horizontal stripes only for the group of participants who believed that horizontal stripes make someone look thinner (*r* = −.24), but not for the group that believed that horizontal stripes make someone look wider (*r* = −.01). That is, the more someone believed that stripes made someone look thin, the more they perceived the striped dress to be thinner compared to the non-stripes dress. However, there was no significant correlation between beliefs and perception for the group of participants who believed that stripes make someone look wider. Additionally, the more participants believed that stripes make someone look wider the more they believed in neuromyths (*r* = .24), but there was no significant correlation with neuromyths for the group of participants who believed that stripes make someone look thinner (*r* = −.01). Furthermore, participants who believed that horizontal stripes make someone look wider would suggest to a friend to buy a non-striped dress to look thinner (*r* = .17), whereas participants who believed that stripes make someone look thinner would suggest a horizontally striped dress (*r* = −.22). Finally, participants who believed that horizontal stripes have a widening effect reported taking more into consideration the possible thinning or widening effect of horizontal stripes when purchasing clothes (*r* = .20).

**Table 3:** Experiment 2 – Intercorrelations among main variables for the combined dataset (Greece, Netherlands, Taiwan).

	*M*	*SD*	1	2	3	4	5	6	7	8	9	10	11	12	13	14
1. PSE	0.97	1.06														
2. Belief horizontal stripes (thin)	27.06	9.83	−.24^**^													
3. Belief horizontal stripes (wide)	71.03	10.39	−.01													
4. Belief horizontal stripes	56.36	22.17	−.04													
5. Neuromyths	45.82	10.53	.01	−.01	.24^***^	−.03										
6. Gender	0.84	0.39	−.03	−.10	−.06	.15^**^	−.11^*^									
7. Age	21.01	4.55	−.03	.03	.09	.05	.22^***^	−.07								
8. Height	166.8	8.07	.06	.03	.13^*^	−.01	.08	−.57^***^	−.01							
9. Weight	61.09	11.35	.04	.02	.11	.04	.05	−.39^***^	.07	.57^***^						
10. BODDS	2.02	0.71	−.02	−.06	.11	.04	.03	.08	.09	−.13^**^	.45^***^					
11. Number of striped clothes	2.91	4.51	.00	−.02	.00	−.03	.10^*^	.05	.10^*^	.04	−.04	−.02				
12. Fashion importance	66.27	22.40	.11^*^	−.10	.02	.05	−.05	.19^***^	−.17^***^	.03	.01	−.05	.07			
13. Outlook importance	68.51	21.01	.04	−.03	−.07	−.05	.06	.00	−.03	.04	−.03	−.03	.04	.42^***^		
14. Stripe consideration	40.95	33.58	−.02	−.04	.20^***^	.32^***^	.08	.11^*^	.03	−.10^*^	.13^**^	.26^***^	.00	.10^*^	.18^***^	
15. Friend advice	49.42	29.95	.11^*^	−.22^*^	−.17^**^	−.45^***^	.08	−.02	.06	−.07	−.03	.07	.05	−.08	.08	−.13^**^

*Notes*. *N* = 419; PSE = point of subjective equality (positive values indicate a thinning perceptual effect); ‘thin’ (*n* = 125) refers to the sub-group of participants who believed that horizontal stripes make someone look thinner; ‘wide’ (*n* = 263) refers to the sub-group of participants who believed that horizontal stripes make someone look wider; *M* = Mean; *SD* = Standard deviation; regarding gender: men = 1, women = 2; BODDS = body size and shape scale. ^*^p < .05; ^**^p < .01; ^***^p < .001.

#### Thinning effect of horizontal stripes.

The results showed that the horizontally striped stimulus was perceived as significantly thinner compared to the non-striped stimulus (PSE = 0.08°, *t* = 12.12, *df* = 422.11, *p* < .001; Cohen’s *d* = 1.18), in line with previous findings [5–7]. The thinning effect was slightly different across countries (PSE_Greece_ = 0.08°; PSE_Netherlands_ = 0.10°; PSE_Taiwan_ = 0.07°), but these differences were not statistically significant (*F*_*2*, 410.1_ = 2.04, *p* = 0.13). These results provided support to the cross-cultural generalizability of the illusion, as both Western and East Asian cultures perceived horizontally striped stimuli as thinner compared to equally wide non-striped stimuli. The thinning effect was also non-significantly different between men and women (*t* = −0.29, *df* = 428.64, *p* = 0.77), *t*hus, in subsequent analyses we pooled together the data across countries and gender.

#### Relation between PSE and self-reported beliefs.

To measure the relation between PSE and participants’ beliefs regarding the effect of horizontal stripes in clothes, we first split participants into two groups to address the trimodal distribution of participants’ beliefs. The first group consisted of participants who believed that horizontal stripes have a thinning effect (*n* = 125), and the second group (*n* = 263) consisted of participants who believed that horizontal stripes have a widening effect. We decided to exclude the group of participants who believed that horizontal stripes have neither a thinning nor a widening effect (*n* = 31), because there was no variation in people’s beliefs in this group (only value ‘50’). Therefore, instead of calculating the correlation between PSE and participants’ beliefs, we tested whether the correlation between participants’ beliefs and PSE was different for each of the two belief groups (thinning, widening). This moderation effect was not described in the preregistration of the experiment, but it seemed appropriate based on the observed distribution of participants’ self-reported beliefs.

The self-reported beliefs were centered based on the mean score of each belief group (i.e., thinning, widening). The results – after controlling for neuromyths, age, gender, height, weight, body size and shape, as well as country – showed a significant interaction between participants’ beliefs and belief group (*t* = 1.95, *df* = 370.48, *p* = 0.051). More specifically, the more participants believed that horizontal stripes in clothes make someone look thinner, the more they perceived the horizontally striped stimulus as thinner, thus strengthening the thinning perceptual effect (*b* = −0.003, *p* = 0.01). However, the effect was non-significant for participants who believed that horizontal stripes make someone look wider (*b* < −0.002, *p* = 0.24). None of the control variables had a significant effect on participants’ PSE. Table 4 presents the results for all predictors and control variables, as well as the interaction between participants’ beliefs and belief group.

**Table 4:** Experiment 2 – Regression coefficients for all predictors and control variables.

	*b*	*SE*	*df*	*t*	*p*
(Intercept)	.084	.023	386.50	3.64	<.001
Beliefs of horizontal stripes	−.003	.001	369.75	−2.58	.01
Belief group	−.001	.008	376.76	−0.07	.94
Neuromyths	<.001	.001	373.24	0.29	.77
Age	<.001	.002	382.07	0.10	.92
Gender	−.004	.020	381.99	−0.22	.83
Height	<.001	.001	375.10	0.13	.90
Weight	<.001	.001	37.04	−0.01	.99
BODDS	−.005	.014	372.34	−0.36	.72
Country (Netherlands)	.026	.017	37.96	1.51	.13
Country (Taiwan)	−.011	.018	369.72	−0.61	.54
Beliefs of horizontal stripes × Belief group	.002	.001	370.48	1.95	.051

*Notes*. Dependent variable is point of subjective equality (PSE); BODDS = body size and shape scale; *SE* = standard error.

**Fig 4:**
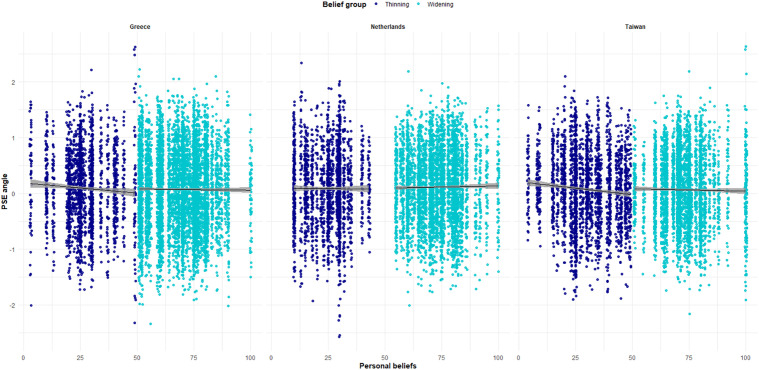
Experiment 2 – Relation between self-reported beliefs and PSE per country and belief group. *Notes*. In dark blue color, the values 0-49 represent a thinning effect with smaller values representing a stronger thinning effect; in light blue color, values 51-100 represent a widening effect with larger values representing a stronger widening effect; participants whose self-reported beliefs had the value ‘50’ (neither believing to a thinning or fattening effect) have been excluded.

Finally, we tested whether the interaction between self-reported beliefs and belief group was different per country (again, only for participants who either believed that horizontal stripes make someone look thin or wide). The results showed that the three-way interaction (self-reported beliefs × belief group × country) was non-significant (*F*_2,372.36_ = 0.27, *p* = 0.77), meaning that the effect of beliefs on size perception generalized across the three countries. Fig 4 illustrates the effect of participants’ beliefs on PSE for each belief group and for each country separately.

## Discussion

### Beliefs regarding the effect of horizontal stripes on the general population

The results of the present work made four main contributions to the extant literature, and the results are summarized in Table 5. First, our data provided support to the belief that horizontal stripes in clothes make someone look wider (Experiments 1 and 2; total *n* = 725). This belief was observed both in Western (Greece, Netherlands) and East Asian (Taiwan) cultures, without significant differences. Interestingly, self-reported beliefs followed a tri-modal distribution. Across both experiments, the majority of participants believed that clothes with horizontal stripes make someone look wider (69.25%), a minority (21.36%) believed that horizontal stripes make someone look thinner, and an even smaller minority believed that horizontal stripes have no effect at all (9.39%). When comparing the general (Experiment 1) to the student population (Experiment 2), a smaller proportion of the general population held the – empirically correct – belief that horizontal stripes have a thinning effect (10.13% vs. 29.83%, for general and student population, respectively). This difference could be explained due to the fact that psychology students might be somewhat familiar with optical illusions, or perhaps more likely, because the belief of the widening effect of horizontal stripes in clothes was positively correlated with age, and participants from the general population were on average older than the students.

**Table 5:** Open research questions and research hypothesis of the present study.

Open research question/Hypothesis	Result
Quantify people’s beliefs regarding the effect of horizontal stripes in clothes.	Experiment 1: Entire sample *Mean* = 64.37 Group: Thinning effect: *Mean* = 34.59 (*n* = 32) Group: Neither thinning nor widening: *Mean* = 50 (*n* = 38) Group: Widening effect: *Mean* = 70.46 (*n* = 246) Experiment 2: Entire sample *Mean* = 56.36 Group: Thinning effect: *Mean* = 27.06 (*n* = 125) Group: Neither thinning nor widening: *Mean* = 50 (*n* = 31) Group: Widening effect: *Mean* = 71.03 (*n* = 263)
What is the correlation between neuromyths with participants’ beliefs on the effect of horizontal stripes in clothes?	Experiment 1: Entire sample *r* = .15 Group: Thinning effect *r* = −.05 Group: Widening effect *r* = .12 Experiment 2: Entire sample *r* = −.03 Group: Thinning effect *r* = −.01 Group: Widening effect *r* = .24
What is the direction and strength of correlation between participants’ beliefs on the perceptual effect of horizontally striped clothes with their visual perception of a horizontally striped stimulus?	The more participants believed that horizontal stripes make someone look thinner, the more they perceived the striped dress as thinner (*r* = −.24). The relation between beliefs and size perception was non-significant for the group believing that horizontal stripes make someone look wider (*r* = −.01).
A horizontally striped dress would be perceived as thinner compared to an identical dress without stripes	Hypothesis confirmed (Cohen’s *d* = 1.18)

### Beliefs regarding the effect of horizontal stripes and neuromyths

Second, for the group of participants who believed that horizontally striped clothes have a widening effect, there was a positive correlation between their beliefs and the belief in neuromyths. At the same time, there was no significant correlation between beliefs and neuromyths for the group of participants who believed that horizontally striped clothes have a thinning effect. In other words, when participants held an erroneous belief regarding the perceptual effect of horizontal stripes, they also tended to hold erroneous beliefs regarding other empirical findings.

### Cross-cultural generalization of the Helmholtz illusion

Third, in the perceptual task, participants perceived a horizontally striped dress as thinner compared to an identical non-striped dress, in line with previous research [5–7]. One might have expected cultural differences regarding the illusion, as participants in Eastern (vs. Western) cultures employ more holistic perceptual strategies [20,21], which enhance sensitivity to background cues that might increase (or decrease) the strength of the illusion [22]. However, the results showed that the thinning effect was similarly observed in both cultures, suggesting that the optical illusion of horizontally striped stimuli being perceived as thinner compared to equally wide non-striped stimuli seems to be cross-culturally generalizable (at least across the two tested cultures).

It has also been suggested that cultural differences in optical illusions might emerge due to differences in the landscape or how technology affects the shape of daily objects (e.g., *carpentered world*, *foreshortening of receding horizontals*, *symbolizing three dimensions in two* hypotheses; [39]). For instance, one previous study [40] tested the cross-cultural generalizability of the Helmholtz illusion across two countries (Scottland, Ghana; *n* = 332), finding that Ghanaian students were somewhat more susceptible to the illusion compared to Scottish students. The difference was attributed to the *foreshortening of receding horizontals* hypothesis [39]. According to this hypothesis, inhabitants of environments with open plains that extend into the distance (e.g., Ghana) are more susceptible to geometrical illusions of lines extended into the distance, compared to inhabitants of environments with restricted open fields, for instance, due to hills or mountains (e.g., Scotland). However, in the same study, the difference was eliminated when tested on a sub-population of students who had received professional training in art or architecture (*n* = 255); that is, in a population that had affinity in working with and interpreting geometrical shapes. The authors speculated that, when exposed to similar training, cultural differences in geometrical illusions diminish. In our present study, a similar explanation could also be applied to the absence of differences between Western and East Asian cultures, as participants of both cultures follow rather similar educational systems that might minimize any perceptual differences.

### Beliefs regarding the effect of horizontal stripes and size perception

Fourth, we found that for the group of participants who believed that horizontal stripes make someone look thinner, participants’ beliefs were positively correlated with their size perception, such that the more someone believed that horizontal stripes in clothes make someone look thinner, the more they perceived the horizontally striped stimulus as thinner compared to the non-striped stimulus. However, for the group of participants who believed that horizontal stripes make someone look wider, participants’ beliefs were not significantly correlated with their size perception (nevertheless, they *still* perceived the horizontally striped stimulus as thinner compared to the non-striped stimulus). In other words, the results highlighted an *asymmetry* in the relation between beliefs and perception, showing that this relation is not straightforward and does not seem to be fully accounted for by a single theoretical framework.

More specifically, regarding the group of participants who believed that stripes have a thinning effect, the results seem to align with evidence supporting cognitive penetration of perception, as demonstrated in other sensory modalities [12,13]. One possible explanation might be provided by predictive coding. According to this framework the brain continuously generates high-level hypotheses about incoming sensory data, sending top-down predictions to lower cortical areas and comparing them against bottom-up signals, minimizing a prediction error [41,42]. Under this framework, the thinning effect of horizontal stripes might arise not only from early orientation-selective neurons (a bottom-up process; [10]) but mainly from prior beliefs about clothing and body shape biasing perceptual inference (a top-down process; [43]).

Regarding the group of participants who believed that stripes have a widening effect, one possible explanation for the non-significant relation between beliefs and perception seems to be provided by feedforward models, according to which perception is largely immune to cognition [28]. For instance, [28] have claimed that cognitive influences on perception are limited or non-existent. That is, perceptual systems are encapsulated, with minimal or no influence from cognitive expectations or beliefs. However, neither of the abovementioned theoretical frameworks fully explain the reasons for observing the asymmetry in the first place. For instance, if a top-down explanation (e.g., predictive coding) was ubiquitous, we should have observed a positive correlation between beliefs and size perception even for the group of participants who believed in a widening effect of stripes – such that the more participants believed that horizontal stripes have a widening effect, the more they should have perceived the striped dress as wider. Similarly, if a bottom-up explanation (e.g., feedforward models) was ubiquitous, we should have observed a non-significant correlation between beliefs and perception regardless of one’s beliefs.

What might be the reasons for the asymmetry? It can be speculated that participants who believe that horizontal stripes have a thinning effect hold more strongly to their beliefs, as it goes against the norm. Counter-normative beliefs might be associated with stronger prior expectations, which could exert greater top-down influence on perception. Instead, participants who believe in a widening effect might hold less strongly to their beliefs, as that belief simply goes with the flow (it is aligned with the normative beliefs), and they might be less invested in or influenced by it. Even though we did not directly collect strength of belief, the results replicated even when we controlled for cultural beliefs (whether participants believed that *other* people believe that stripes have a thinning or widening effect) as well as the difference between participants’ own beliefs and the perceived cultural beliefs (a proxy on how strongly participants’ beliefs deviated from the norm). An alternative explanation is that the asymmetry reflects individual differences in sensitivity to one’s own perceptual experience or in the tendency to revise beliefs in response to counter-evidence. However, as the present study did not include measures of belief-updating or metacognitive sensitivity, these possibilities cannot be empirically evaluated with the current data and constitute an important direction for future research.

In any respect, the asymmetrical effect – the fact the beliefs were correlated with size perception only for participants who believed that stripes in clothes make someone look thinner, but not for the group of participants who believed that horizontal stripes make someone look wider – was observed across both Western and East Asian cultures, after controlling for participants’ gender, age, height, weight, body size and type, and neuromyth beliefs. These results suggest that the phenomenon seems to be culturally generalizable, holding even when controlling for a large number of possible confounding variables.

### Limitations and suggestions for future research

The present study had also some limitations. First, even though we did not hypothesize that participants’ beliefs regarding the effect of horizontal stripes will follow a trimodal distribution, such a distribution might have been expected based on the scale used to measure participants’ beliefs. To overcome such distribution, we split participants into two groups, something that lead to excluding participants who did not believe in either a thinning or widening effect – a suboptimal solution. To avoid such statistical shortcomings, future studies could experiment with different response formats, avoiding trimodal distributions. However, based on the results of the present study, future studies should probably expect that participants’ beliefs will likely be strongly unbalanced.

Second, the effect of participants’ beliefs on size perception was based on a student sample and we cannot be certain whether the results would generalize to the general population. This is because the student sample was based on Psychology students who, compared to the general population, were younger and had greater knowledge of psychological facts, such as neuromyths or beliefs regarding the effect of stripes on clothes. Future studies could replicate the effects of Experiment 2 on the general population. Given the large imbalance between participants who believed in a thinning vs. widening effect of horizontal stripes in the general population, we suggest that the required sample size needs to be substantially larger compared to the present study.

## Conclusion

The present study provided empirical evidence to the popular belief that horizontal stripes in clothes make someone look wider. Even though the majority of the population self-reported that horizontal stripes have a widening effect, a substantial minority held the empirically correct belief that horizontal stripes have a thinning effect. We further replicated the thinning effect of horizontally stripes stimuli compared to equally wide non-striped stimuli (Helmholtz illusion) in a behavioral task in a cross-cultural sample, including Western (Greece, Netherlands) and East Asian (Taiwan) cultures. Additionally, our results showed that one’s susceptibility to the illusion was related to their beliefs regarding the effect of horizontal stripes, but this relation was asymmetrical. That is, the more someone believed that horizontal stripes have a thinning effect, the more they perceived a horizontally striped stimulus as thinner compared to an equally wide non-striped stimulus. However, this relation was non-significant for participants who believed that horizontal stripes have a widening effect. This relation was similarly observed across cultures suggesting that the effect can be cross-culturally generalizable.
