# Supplementary material for: When stripes in clothes deceive: Cross-cultural examination of perceptual and belief discrepancies about horizontal stripes in clothes
Source: PLoS One. 2026 Apr 30;21(4):e0347495. doi: 10.1371/journal.pone.0347495 (PMC13132240; doi:10.1371/journal.pone.0347495)
Supplement: Peer review history

# Peer review: Decision Letter 0

**DOI:** 10.1371/journal.pone.0347495.r001

**Authors:** Steve Zimmerman

29 Jan 2026

PONE-D-25-63295

When stripes in clothes deceive: Cross-cultural examination of perceptual and belief discrepancies about horizontal stripes in clothes

PLOS One

Dear Dr. Koutsoumpis,

Thank you for submitting your manuscript to PLOS ONE. After careful consideration, we feel that it has merit but does not fully meet PLOS ONE’s publication criteria as it currently stands. Therefore, we invite you to submit a revised version of the manuscript that addresses the points raised during the review process.

Please note that we have only been able to secure a single reviewer to assess your manuscript. We are issuing a decision on your manuscript at this point to prevent further delays in the evaluation of your manuscript. Please be aware that the editor who handles your revised manuscript might find it necessary to invite additional reviewers to assess this work once the revised manuscript is submitted.

The reviewer's comments can be found below. Could you please revise the manuscript to carefully address the concerns raised?

Please submit your revised manuscript by Mar 14 2026 11:59PM. If you will need more time than this to complete your revisions, please reply to this message or contact the journal office at plosone@plos.org. When you're ready to submit your revision, log on to https://www.editorialmanager.com/pone/ and select the 'Submissions Needing Revision' folder to locate your manuscript file.. When you're ready to submit your revision, log on to https://www.editorialmanager.com/pone/ and select the 'Submissions Needing Revision' folder to locate your manuscript file.

Please include the following items when submitting your revised manuscript:

- A letter that responds to each point raised by the academic editor and reviewer(s). You should upload this letter as a separate file labeled 'Response to Reviewers'.
- A marked-up copy of your manuscript that highlights changes made to the original version. You should upload this as a separate file labeled 'Revised Manuscript with Track Changes'.
- An unmarked version of your revised paper without tracked changes. You should upload this as a separate file labeled 'Manuscript'.

-->If you would like to make changes to your financial disclosure, please include your updated statement in your cover letter. Guidelines for resubmitting your figure files are available below the reviewer comments at the end of this letter.

If applicable, we recommend that you deposit your laboratory protocols in protocols.io to enhance the reproducibility of your results. Protocols.io assigns your protocol its own identifier (DOI) so that it can be cited independently in the future. For instructions see: https://journals.plos.org/plosone/s/submission-guidelines#loc-laboratory-protocols. Additionally, PLOS ONE offers an option for publishing peer-reviewed Lab Protocol articles, which describe protocols hosted on protocols.io. Read more information on sharing protocols at . Additionally, PLOS ONE offers an option for publishing peer-reviewed Lab Protocol articles, which describe protocols hosted on protocols.io. Read more information on sharing protocols at https://plos.org/protocols?utm_medium=editorial-email&utm_source=authorletters&utm_campaign=protocols..

We look forward to receiving your revised manuscript.

Kind regards,

Steve Zimmerman, PhD

Senior Editor, PLOS One

Journal Requirements:

When submitting your revision, we need you to address these additional requirements.

1. Please ensure that your manuscript meets PLOS ONE's style requirements, including those for file naming. The PLOS ONE style templates can be found at

https://journals.plos.org/plosone/s/file?id=wjVg/PLOSOne_formatting_sample_main_body.pdf and https://journals.plos.org/plosone/s/file?id=ba62/PLOSOne_formatting_sample_title_authors_affiliations.pdf

2. Please provide additional details regarding participant consent. In the ethics statement in the Methods and online submission information, please ensure that you have specified what type you obtained (for instance, written or verbal, and if verbal, how it was documented and witnessed). If your study included minors, state whether you obtained consent from parents or guardians. If the need for consent was waived by the ethics committee, please include this information.

3. Please note that your Data Availability Statement is currently missing the repository name and/or the DOI/accession number of each dataset. If your manuscript is accepted for publication, you will be asked to provide these details on a very short timeline. We therefore suggest that you provide this information now, though we will not hold up the peer review process if you are unable.

4. Please include your full ethics statement in the ‘Methods’ section of your manuscript file. In your statement, please include the full name of the IRB or ethics committee who approved or waived your study, as well as whether or not you obtained informed written or verbal consent. If consent was waived for your study, please include this information in your statement as well.

5. We note that Figure 1 in your submission may contain copyrighted images. All PLOS content is published under the Creative Commons Attribution License (CC BY 4.0), which means that the manuscript, images, and Supporting Information files will be freely available online, and any third party is permitted to access, download, copy, distribute, and use these materials in any way, even commercially, with proper attribution. For more information, see our copyright guidelines: http://journals.plos.org/plosone/s/licenses-and-copyright.

We require you to either (1) present written permission from the copyright holder to publish these figures specifically under the CC BY 4.0 license, or (2) remove the figures from your submission:

1. You may seek permission from the original copyright holder of Figure 1 to publish the content specifically under the CC BY 4.0 license.

We recommend that you contact the original copyright holder with the Content Permission Form (http://journals.plos.org/plosone/s/file?id=7c09/content-permission-form.pdf) and the following text:

“I request permission for the open-access journal PLOS ONE to publish XXX under the Creative Commons Attribution License (CCAL) CC BY 4.0 (http://creativecommons.org/licenses/by/4.0/). Please be aware that this license allows unrestricted use and distribution, even commercially, by third parties. Please reply and provide explicit written permission to publish XXX under a CC BY license and complete the attached form.”

Please upload the completed Content Permission Form or other proof of granted permissions as an "Other" file with your submission.

In the figure caption of the copyrighted figure, please include the following text: “Reprinted from [ref] under a CC BY license, with permission from [name of publisher], original copyright [original copyright year].”

2. If you are unable to obtain permission from the original copyright holder to publish these figures under the CC BY 4.0 license or if the copyright holder’s requirements are incompatible with the CC BY 4.0 license, please either i) remove the figure or ii) supply a replacement figure that complies with the CC BY 4.0 license. Please check copyright information on all replacement figures and update the figure caption with source information. If applicable, please specify in the figure caption text when a figure is similar but not identical to the original image and is therefore for illustrative purposes only.

If the reviewer comments include a recommendation to cite specific previously published works, please review and evaluate these publications to determine whether they are relevant and should be cited. There is no requirement to cite these works unless the editor has indicated otherwise.

Please review your reference list to ensure that it is complete and correct. If you have cited papers that have been retracted, please include the rationale for doing so in the manuscript text, or remove these references and replace them with relevant current references. Any changes to the reference list should be mentioned in the rebuttal letter that accompanies your revised manuscript. If you need to cite a retracted article, indicate the article’s retracted status in the References list and also include a citation and full reference for the retraction notice.

[Note: HTML markup is below. Please do not edit.]

Reviewers' comments:

Reviewer's Responses to Questions

**Comments to the Author**

1. Is the manuscript technically sound, and do the data support the conclusions?

The manuscript must describe a technically sound piece of scientific research with data that supports the conclusions. Experiments must have been conducted rigorously, with appropriate controls, replication, and sample sizes. The conclusions must be drawn appropriately based on the data presented.

Reviewer #1: Yes

**********

2. Has the statistical analysis been performed appropriately and rigorously?

Reviewer #1: I Don't Know

**********

3. Have the authors made all data underlying the findings in their manuscript fully available?

The PLOS Data policy requires authors to make all data underlying the findings described in their manuscript fully available without restriction, with rare exception (please refer to the Data Availability Statement in the manuscript PDF file). The data should be provided as part of the manuscript or its supporting information, or deposited to a public repository. For example, in addition to summary statistics, the data points behind means, medians and variance measures should be available. If there are restrictions on publicly sharing data—e.g. participant privacy or use of data from a third party—those must be specified.requires authors to make all data underlying the findings described in their manuscript fully available without restriction, with rare exception (please refer to the Data Availability Statement in the manuscript PDF file). The data should be provided as part of the manuscript or its supporting information, or deposited to a public repository. For example, in addition to summary statistics, the data points behind means, medians and variance measures should be available. If there are restrictions on publicly sharing data—e.g. participant privacy or use of data from a third party—those must be specified.

Reviewer #1: Yes

**********

4. Is the manuscript presented in an intelligible fashion and written in standard English?

PLOS ONE does not copyedit accepted manuscripts, so the language in submitted articles must be clear, correct, and unambiguous. Any typographical or grammatical errors should be corrected at revision, so please note any specific errors here.

Reviewer #1: Yes

**********

5. Review Comments to the Author

Please use the space provided to explain your answers to the questions above. You may also include additional comments for the author, including concerns about dual publication, research ethics, or publication ethics. (Please upload your review as an attachment if it exceeds 20,000 characters)

Reviewer #1: Conducting an Internet survey with participants from Greece and Netherlands, and a lab experiment with participants from Greece, Netherlands, and Taiwan, the authors showed that most peoples of those countries have the popular belief that horizontal stripes in clothes make the wearer look wider, and yet the perceptual task demonstrated that most participants exhibited a thinning effect of horizontal stripes in clothes. Moreover, the authors found that the more participants believed that horizontal stripes have a thinning effect, the more they perceived a horizontally striped stimulus as thinner, but this correlation was non-significant for participants who believed that horizontal stripes have a widening effect.

The design of the experiments are good, and the findings are interesting. My greatest concern is the concepts of individualistic culture vs. collectivistic culture, which are somewhat prejudiced against the latter. As a basis for this distinction, the authors cited Papers 16-21, but they hardly rely on those concepts. Calling Taiwanese collectivists is judgmental, even somewhat insulting. Recently some researchers have questioned the notion that Western culture is individualistic and Eastern culture is collectivistic. Please delete "individualistic" vs. "collectivistic", and use Western vs. Eastern, or analytic vs. holistic, instead.

Details of the statistical analyses are not very clear to me. Am I correct in assuming that the authors essentially conducted multiple regression analyses?

The asymmetry of correlations between beliefs and actual size perception is the most interesting part of the study. The authors gave much thought to why the asymmetry occured, but unable to solve the mystery. I do have an idea. Perhaps the asymmetry arises because of individual differences in sensitivity to one's own perception and willingness to revise one's own beliefs. Thanks to abundant information on mass media and the Internet, we all are exposed to the fashion industry's "common knowledge" that horizontal stripes in clothes make the wearer look wider. But some people are more sensitive to their own perceptions than other people. Those sensitive people eventually notice through their own experience that horizontal stripes actually make the wearer look thinner, and revise their belief accordingly. People who are not so sensitive to their own perceptions may experience the same thinning effect of horizontal stripes, but they dismiss the experience and fail to revise their belief. This conjecture is supported by the authors' finding (Lines 423-426) that only the participants who believed horizontal stripes' widening effect exhibited a significant correlation with their belief in neuromyths, but not those who believed horizontal stripes' thinning effect; the former group fail to revise their belief in neuromyths even after they experienced counter-evidence.

Minor corrections:

Line 20: "was related" should be "were related".

Line 87-89: "(i.e., ... widening) effect" is totally redundant and should be deleted.

Line 170: "at the 25th" should be "on the 25th".

Line 275: "they" is grammartically wrong and should be deleted.

Line 327: "dimply" should be "dimly".

Line 380: I am not sure what the authors mean by "were centered by country"etc. Do you mean "were standardized by country"? Please clarify.

Line 540: "extent" should be "extend".

**********

6. PLOS authors have the option to publish the peer review history of their article (what does this mean?). If published, this will include your full peer review and any attached files.). If published, this will include your full peer review and any attached files.

If you choose “no”, your identity will remain anonymous but your review may still be made public.

**Do you want your identity to be public for this peer review?** For information about this choice, including consent withdrawal, please see our For information about this choice, including consent withdrawal, please see our Privacy Policy..

Reviewer #1: **Yes:**Kazunori MorikawaKazunori Morikawa

**********

[NOTE: If reviewer comments were submitted as an attachment file, they will be attached to this email and accessible via the submission site. Please log into your account, locate the manuscript record, and check for the action link "View Attachments". If this link does not appear, there are no attachment files.]

To ensure your figures meet our technical requirements, please review our figure guidelines: https://journals.plos.org/plosone/s/figures

You may also use PLOS’s free figure tool, NAAS, to help you prepare publication quality figures: https://journals.plos.org/plosone/s/figures#loc-tools-for-figure-preparation.

NAAS will assess whether your figures meet our technical requirements by comparing each figure against our figure specifications.

# Author comment: Author response to Decision Letter 1

**DOI:** 10.1371/journal.pone.0347495.r002

9 Feb 2026

Comment 1: The design of the experiments are good, and the findings are interesting. My greatest concern is the concepts of individualistic culture vs. collectivistic culture, which are somewhat prejudiced against the latter. As a basis for this distinction, the authors cited Papers 16-21, but they hardly rely on those concepts. Calling Taiwanese collectivists is judgmental, even somewhat insulting. Recently some researchers have questioned the notion that Western culture is individualistic and Eastern culture is collectivistic. Please delete "individualistic" vs. "collectivistic", and use Western vs. Eastern, or analytic vs. holistic, instead.

Response: We would like to thank you for pointing out this important language issue. Naturally, our intention is not to use discriminatory language. We have updated our manuscript accordingly, replacing the terms ‘individualistic’ and ‘collectivistic’ with ‘Western’ and ‘East Asian’, accordingly.

Comment 2: Details of the statistical analyses are not very clear to me. Am I correct in assuming that the authors essentially conducted multiple regression analyses?

Response: Thank you for this important point. We did not conduct multiple regression analyses in the traditional sense. On page 18, we now explain in more detail that we used a linear mixed-effects (multilevel) regression model: “Unlike a standard multiple regression analysis, which assumes that all observations are independent and belong to a single level of analysis, in our data this assumption was violated. This was because the repeated PSE measurements were nested within participants, and participants were nested within countries. Therefore, we employed a linear mixed-effects (multilevel) model which explicitly accounts for this hierarchical structure by modeling both fixed effects (e.g., beliefs, belief group, and control variables) and random effects associated with higher-level units (e.g., participants and country).”

Comment 3: The asymmetry of correlations between beliefs and actual size perception is the most interesting part of the study. The authors gave much thought to why the asymmetry occured, but unable to solve the mystery. I do have an idea. Perhaps the asymmetry arises because of individual differences in sensitivity to one's own perception and willingness to revise one's own beliefs. Thanks to abundant information on mass media and the Internet, we all are exposed to the fashion industry's "common knowledge" that horizontal stripes in clothes make the wearer look wider. But some people are more sensitive to their own perceptions than other people. Those sensitive people eventually notice through their own experience that horizontal stripes actually make the wearer look thinner, and revise their belief accordingly. People who are not so sensitive to their own perceptions may experience the same thinning effect of horizontal stripes, but they dismiss the experience and fail to revise their belief. This conjecture is supported by the authors' finding (Lines 423-426) that only the participants who believed horizontal stripes' widening effect exhibited a significant correlation with their belief in neuromyths, but not those who believed horizontal stripes' thinning effect; the former group fail to revise their belief in neuromyths even after they experienced counter-evidence.

Response: We would like to thank you for this thoughtful and stimulating suggestion. The proposed explanation—that the observed asymmetry may arise from individual differences in sensitivity to one’s own perceptual experience and the willingness to revise one’s beliefs—is indeed plausible and theoretically interesting. We fully agree that such mechanisms could contribute to why participants who hold counter normative beliefs (i.e., that horizontal stripes have a thinning effect) show a stronger alignment between their beliefs and perceptual judgments, whereas participants endorsing the culturally normative belief (i.e., widening effect) do not.

At the same time – just like the speculation we put forward in the original manuscript – is not directly testable with the collected dataset. We did not include measures that would allow us to assess individuals’ sensitivity to their perceptual experience, their tendency to update beliefs in light of counter evidence, or the strength with which they hold these beliefs. For this reason, we are not in a position to disentangle between alternative interpretations.

We have now revised the manuscript to clarify this point explicitly. Our manuscript now reads on page 30: “An alternative explanation is that the asymmetry reflects individual differences in sensitivity to one’s own perceptual experience or in the tendency to revise beliefs in response to counter evidence. However, as the present study did not include measures of belief updating or metacognitive sensitivity, these possibilities cannot be empirically evaluated with the current data and constitute an important direction for future research.”

Comment 4: Line 380: I am not sure what the authors mean by "were centered by country" etc. Do you mean "were standardized by country"? Please clarify

Response: Thank you for this comment. In brief, centering means subtracting each value from the mean (such that the variance, standard deviation, and overall distribution of the centered scores remain unchanged relative to the original data), whereas standardization further divides this value with the standard deviation. To make this clearer also for the readers, we added the following text on page 19: “Before entering the linear mixed-effects model, all control and predictor variables were mean-centered based on their relevant groups. Specifically, neuromyths and age were centered within country by subtracting each country’s mean from individual scores. Height, weight, and BODDS were centered within country and gender, such that values reflected deviations from the mean of participants of the same gender within the same country. Self-reported beliefs on the effect of horizontal stripes were centered per country and belief group (see below), so that scores represented deviations from the corresponding group mean. We applied centering (rather than standardization) to remove between-country mean differences while preserving the original metric and variance of the measures for interpretability.”

Minor Comments: Line 20: "was related" should be "were related"; Line 87-89: "(i.e., ... widening) effect" is totally redundant and should be deleted; Line 170: "at the 25th" should be "on the 25th"; Line 275: "they" is grammartically wrong and should be deleted; Line 327: "dimply" should be "dimly"; Line 540: "extent" should be "extend".

Response: Thank you for pointing out these language mistakes. We have updated our manuscript accordingly.

# Peer review: Decision Letter 1

**DOI:** 10.1371/journal.pone.0347495.r003

**Authors:** Giovanni Ottoboni

1 Apr 2026

When stripes in clothes deceive: Cross-cultural examination of perceptual and belief discrepancies about horizontal stripes in clothes

PONE-D-25-63295R1

Dear Dr. Koutsoumpis,

We’re pleased to inform you that your manuscript has been judged scientifically suitable for publication and will be formally accepted for publication once it meets all outstanding technical requirements.

Within one week, you’ll receive an e-mail detailing the required amendments. When these have been addressed, you’ll receive a formal acceptance letter and your manuscript will be scheduled for publication.

An invoice will be generated when your article is formally accepted. Please note, if your institution has a publishing partnership with PLOS and your article meets the relevant criteria, all or part of your publication costs will be covered. Please make sure your user information is up-to-date by logging into Editorial Manager at Editorial Manager® and clicking the ‘Update My Information' link at the top of the page. For questions related to billing, please contact  and clicking the ‘Update My Information' link at the top of the page. For questions related to billing, please contact billing support..

If your institution or institutions have a press office, please notify them about your upcoming paper to help maximize its impact. If they’ll be preparing press materials, please inform our press team as soon as possible -- no later than 48 hours after receiving the formal acceptance. Your manuscript will remain under strict press embargo until 2 pm Eastern Time on the date of publication. For more information, please contact onepress@plos.org.

Kind regards,

Giovanni Ottoboni, Psy, PhD

Academic Editor

PLOS One

Additional Editor Comments (optional):

Reviewers' comments:

Reviewer's Responses to Questions

**Comments to the Author**

1. If the authors have adequately addressed your comments raised in a previous round of review and you feel that this manuscript is now acceptable for publication, you may indicate that here to bypass the “Comments to the Author” section, enter your conflict of interest statement in the “Confidential to Editor” section, and submit your "Accept" recommendation.

Reviewer #1: All comments have been addressed

**********

2. Is the manuscript technically sound, and do the data support the conclusions?

The manuscript must describe a technically sound piece of scientific research with data that supports the conclusions. Experiments must have been conducted rigorously, with appropriate controls, replication, and sample sizes. The conclusions must be drawn appropriately based on the data presented.

Reviewer #1: (No Response)

**********

3. Has the statistical analysis been performed appropriately and rigorously?

Reviewer #1: (No Response)

**********

4. Have the authors made all data underlying the findings in their manuscript fully available?

The PLOS Data policy requires authors to make all data underlying the findings described in their manuscript fully available without restriction, with rare exception (please refer to the Data Availability Statement in the manuscript PDF file). The data should be provided as part of the manuscript or its supporting information, or deposited to a public repository. For example, in addition to summary statistics, the data points behind means, medians and variance measures should be available. If there are restrictions on publicly sharing data—e.g. participant privacy or use of data from a third party—those must be specified.requires authors to make all data underlying the findings described in their manuscript fully available without restriction, with rare exception (please refer to the Data Availability Statement in the manuscript PDF file). The data should be provided as part of the manuscript or its supporting information, or deposited to a public repository. For example, in addition to summary statistics, the data points behind means, medians and variance measures should be available. If there are restrictions on publicly sharing data—e.g. participant privacy or use of data from a third party—those must be specified.

Reviewer #1: (No Response)

**********

5. Is the manuscript presented in an intelligible fashion and written in standard English?

PLOS ONE does not copyedit accepted manuscripts, so the language in submitted articles must be clear, correct, and unambiguous. Any typographical or grammatical errors should be corrected at revision, so please note any specific errors here.

Reviewer #1: (No Response)

**********

6. Review Comments to the Author

Please use the space provided to explain your answers to the questions above. You may also include additional comments for the author, including concerns about dual publication, research ethics, or publication ethics. (Please upload your review as an attachment if it exceeds 20,000 characters)

Reviewer #1: (No Response)

**********

7. PLOS authors have the option to publish the peer review history of their article (what does this mean?). If published, this will include your full peer review and any attached files.). If published, this will include your full peer review and any attached files.

If you choose “no”, your identity will remain anonymous but your review may still be made public.

**Do you want your identity to be public for this peer review?** For information about this choice, including consent withdrawal, please see our For information about this choice, including consent withdrawal, please see our Privacy Policy..

Reviewer #1: **Yes:**Kazunori MorikawaKazunori Morikawa

**********

# Editor report: Acceptance letter

**DOI:** 10.1371/journal.pone.0347495.r004

**Authors:** Giovanni Ottoboni

PONE-D-25-63295R1

PLOS One

Dear Dr. Koutsoumpis,

I'm pleased to inform you that your manuscript has been deemed suitable for publication in PLOS One. Congratulations! Your manuscript is now being handed over to our production team.

At this stage, our production department will prepare your paper for publication. This includes ensuring the following:

* All references, tables, and figures are properly cited

* All relevant supporting information is included in the manuscript submission,

* There are no issues that prevent the paper from being properly typeset

You will receive further instructions from the production team, including instructions on how to review your proof when it is ready. Please keep in mind that we are working through a large volume of accepted articles, so please give us a few days to review your paper and let you know the next and final steps.

Lastly, if your institution or institutions have a press office, please let them know about your upcoming paper now to help maximize its impact. If they'll be preparing press materials, please inform our press team within the next 48 hours. Your manuscript will remain under strict press embargo until 2 pm Eastern Time on the date of publication. For more information, please contact onepress@plos.org.

You will receive an invoice from PLOS for your publication fee after your manuscript has reached the completed accept phase. If you receive an email requesting payment before acceptance or for any other service, this may be a phishing scheme. Learn how to identify phishing emails and protect your accounts at https://explore.plos.org/phishing.

If we can help with anything else, please email us at customercare@plos.org.

Thank you for submitting your work to PLOS ONE and supporting open access.

Kind regards,

PLOS ONE Editorial Office Staff

on behalf of

Professor Giovanni Ottoboni

Academic Editor

PLOS One
